# Metabolic health and its association with lifestyle habits according to nutritional status in Chile: A cross-sectional study from the National Health Survey 2016-2017

**DOI:** 10.1371/journal.pone.0236451

**Published:** 2020-07-22

**Authors:** Rodrigo Fernández-Verdejo, José Luis Moya-Osorio, Eduardo Fuentes-López, Jose E. Galgani

**Affiliations:** 1 Carrera de Nutrición y Dietética, Departamento de Ciencias de la Salud, Facultad de Medicina, Pontificia Universidad Católica de Chile, Santiago, Chile; 2 Carrera de Fonoaudiología, Departamento de Ciencias de la Salud, Facultad de Medicina, Pontificia Universidad Católica de Chile, Santiago, Chile; 3 Departamento de Nutrición, Diabetes y Metabolismo, Facultad de Medicina, Pontificia Universidad Católica de Chile, Santiago, Chile; Universitat de les Illes Balears, SPAIN

## Abstract

**Background:**

Lifestyle habits associate with metabolic health in overall populations. Whether such association is similar among subjects with a different nutritional status has been less studied. We aimed to (i) determine the prevalence of metabolic phenotypes in Chile, and (ii) determine the association between lifestyle habits and metabolic health according to the nutritional status.

**Methods:**

The National Health Survey of Chile 2016–2017 was analyzed. A metabolically unhealthy phenotype was defined as manifesting ≥3 of the following risk factors: elevated blood pressure, elevated triglycerides, elevated glucose, elevated waist circumference, or reduced high-density lipoprotein cholesterol. Individuals manifesting <2 risk factors were considered as healthy. The nutritional status was defined as normal weight (18.5 to <25 kg/m^2^), overweight (25 to <30 kg/m^2^) or obesity (≥30 kg/m^2^). Questionnaires were used to estimate smoking habits, alcohol intake, sedentary behavior, moderate-vigorous physical activity, fruits/vegetables consumption, and fish/seafood consumption. The association (odds ratio [95%CI]) between lifestyle habits and metabolic health was determined within each nutritional status, adjusting for age, sex, BMI (in kg/m^2^), and education.

**Results:**

The prevalence of a metabolically unhealthy phenotype was 36% in the overall sample. Such a prevalence was 7%, 33% and 58% among subjects with normal weight, overweight and obesity, respectively. In subjects with normal weight, the highest quartile of fruits/vegetables consumption was associated with reduced odds of having a metabolically unhealthy phenotype (0.09 [0.01–0.48]). In subjects with obesity, the highest quartile of moderate-vigorous physical activity was associated with reduced odds of having a metabolically unhealthy phenotype (0.29 [0.09–0.91]).

**Conclusion:**

One third of the Chilean population manifests an unhealthy phenotype. We identified associations between lifestyle habits and metabolic health that are specific to the nutritional status. Thus, emphasizing fruits/vegetables consumption in subjects with normal weight, and physical activity in subjects with obesity, may maximize the benefits of public health interventions.

## Introduction

Metabolic health is determined by the presence of metabolic risk factors of chronic non-communicable diseases–largely heart disease, stroke, cancer, chronic respiratory disease, and diabetes [1]. Nevertheless, there is no universal definition for a metabolically unhealthy phenotype, and different definitions have therefore been used [2–6]. Defining metabolically unhealthy subjects as those afflicted with the metabolic syndrome represents one alternative [2,6,7]. Thus, subjects may be classified as metabolically unhealthy if they manifested 3 or more of the following risk factors: elevated blood pressure, elevated circulating triglycerides, elevated fasting glucose, elevated waist circumference, or reduced high-density lipoprotein cholesterol (HDL-C) [8]. Various criteria have also been used to classify subjects as metabolically healthy, often considering as healthy those subjects manifesting up to 2 risk factors [2,3].

The prevalence of a metabolically unhealthy phenotype relates directly with the body mass index (BMI) [7,9–12]. Thus, only 4–9% of subjects with normal weight classify as metabolically unhealthy, whereas the prevalence reaches 54–63% among subjects with obesity [7,9–12]. Importantly, a metabolically unhealthy phenotype associates with an increased risk of cardiovascular events and all-cause mortality, independently of BMI [13]. This observation highlights the relevance of identifying predisposing factors other than excess body weight, as previously suggested [14].

Lifestyle habits appear as relevant candidates, and are thus the focus of public health recommendations [1]. A meta-analysis showed a higher prevalence of a metabolically unhealthy phenotype in former/current smokers and alcohol consumers compared to never-smokers and teetotalers, respectively [2]. The result was observed both in subjects with normal weight and in those with overweight/obesity. A recent study revealed that physical activity, sedentary time, and smoking habits, associated with metabolic health [12]. This latter study included subjects of all nutritional statuses, and associations were adjusted for BMI. Although this adjustment removes the influence of BMI, it may bias results towards the most prevalent nutritional status. Thus, results in the overall population may reflect the most prevalent group (e.g. overweight), potentially missing associations in minorities (e.g. normal weight). Previous reports have shed light upon associations between lifestyle habits and metabolic health that are specific to the nutritional status. In Korea, smoking was associated with a metabolically unhealthy phenotype among subjects with obesity, but not among subjects with normal weight. In contrast, physical activity was associated with a healthy phenotype only among subjects with normal weight [15]. In Spain, physical activity was associated with a healthy phenotype among subjects with obesity, but not among subjects with normal weight [16]. In the USA, physical activity was associated with a healthy phenotype among subjects with normal weight or overweight, but not in those with obesity [17]. These observations suggest associations between lifestyle habits and metabolic health that are specific to the nutritional status and the population studied.

Identifying the most influential habits for each nutritional status in specific populations would help to personalize interventions. Herein we studied the Chilean population. Chile is a member of the Organization for Economic Cooperation and Development (OECD), an international organization that includes countries with advanced economies. Chile and Mexico are the only Latin American members. Of note, among the OECD members, Chile has the highest income inequality–according to the Gini coefficient–, and the third highest out-of-pocket health spending [18,19]. Chile also has the second highest prevalence of daily smokers (24.5%), and the highest prevalence of overweight/obesity (74.2%) [20,21]. Thus, despite Chile being a developed Latin American country, it has more serious health issues than other developed countries worldwide.

The National Health Survey of Chile 2016–2017 collected information about nutritional status, metabolic health, and lifestyle habits (smoking, alcohol intake, sedentary behavior, moderate-vigorous physical activity, fruits/vegetables consumption, and fish/seafood consumption). We hypothesized that there are associations between lifestyle habits and metabolic health that are specific to certain nutritional statuses. Our aims were: (i) to determine the prevalence of metabolic phenotypes in Chile, and (ii) to determine the association between metabolic health and lifestyle habits among subjects with normal weight, overweight, or obesity in Chile.

## Methods

### Database

This research used data from the Surveys of Health for epidemiologic surveillance by the Public Health Subsecretary of Chile, but our findings do not compromise such Institution. The protocols and written informed consent for the National Health Survey of Chile 2016–2017 were approved by the Scientific Ethics Committee of the Faculty of Medicine of Pontificia Universidad Católica de Chile (CEC-MedUC, project number 16–019) and were in accordance with the Declaration of Helsinki.

We used the STROBE methodology for reporting our study (Checklist in S1 File). In this cross-sectional study, we analyzed data of the National Health Survey of Chile 2016–2017, which was conducted between August 2016 and March 2017. A detailed description of the survey’s methodology has been published elsewhere [22]. Briefly, the National Health Survey was a cross-sectional household survey that included 6,233 participants who were 15 years old or older. The sampling method was stratified and multistage. Thirty strata were considered, which represented urban and rural areas of 15 geographical regions. In the multistage sampling, selection was based on counties as the primary sampling units, then households within counties, and finally one participant from selected households. Sampling weights from the survey accounted for differences in selection probability and non-response rates, and the post-stratification adjustment allowed to expand the sample to the estimated population in Chile. To select participants for the current analyses, we considered the following eligibility criteria: (i) 18 to <65 years old, (ii) BMI ≥18.5 kg/m^2^, and (iii) complete data for the risk factors used to diagnose metabolic syndrome.

### Metabolic phenotype

We used the harmonized definition of metabolic syndrome [8], but considering a cutoff for waist circumference specific for Chilean population [23]. Thus, subjects classified as metabolically unhealthy (Unhealthy) if they manifested ≥3 of the following risk factors: waist circumference ≥91 cm for men, or ≥83 cm for women; circulating triglycerides ≥150 mg/dL; HDL-C <40 mg/dL for men, or <50 mg/dL for women, or under drug treatment for cholesterol control; systolic blood pressure ≥130 mmHg, or diastolic blood pressure ≥85 mmHg, or under antihypertensive drug treatment; and circulating glucose ≥100 mg/dL or under glucose-lowering drug treatment. Individuals with up to 1 risk factor were considered as metabolically healthy (Healthy). Those with 2 risk factors were considered as having an intermediate phenotype, and were not considered for the main analyses of the association between lifestyle habits and metabolic health. Subjects were also classified as having normal weight (BMI = 18.5 to <25.0 kg/m^2^), overweight (BMI = 25.0 to <30.0 kg/m^2^), or obesity (BMI ≥ 30.0 kg/m^2^).

### Blood samples, anthropometry, education, and lifestyle habits

Trained nurses obtained the blood samples and conducted the anthropometric measurements. Details of these procedures were published elsewhere [22]. Education was categorized as <8 years, 8–12 years, or >12 years of education. Lifestyle habits were obtained through questionnaires. Smoking habits were categorized as current, former or never. For alcohol intake, the short version of the Alcohol Use Disorders Identification Test (AUDIT-C) was applied [24]. Alcohol intake was then categorized according to tertiles of the AUDIT-C score. The Global Physical Activity Questionnaire was applied to determine levels of moderate-vigorous physical activity (in MET×min/wk) and of sedentary behavior (in min/d) [25]; for analyses, these two continuous variables were categorized into quartiles. Consumption of fruits and vegetables was obtained through questionnaires about the amount–in 80-g portions–and frequency of consumption in a standard week; fruits/vegetables consumption was then categorized into quartiles. Consumption of fish/seafood was determined through a single question about the frequency of consumption; four alternatives of response were allowed: <1 time/month, 1 to <3 times/month, 4 times/month, or >4 times/month. Subjects with missing data for a certain lifestyle habit were excluded from the analyses encompassing that lifestyle habit.

### Analyses

For continuous variables, we computed upper (Q3 + 3 × [Q3 –Q1]) and lower (Q1–3 × [Q3 –Q1]) limits to identify extreme outliers. Those outliers were excluded, as it has been done in previous versions of the Chilean National Health Survey [26]. Data for continuous variables (age, height, weight, BMI) were expressed as mean [95% confidence intervals; 95% CI] (n applying sampling weights). Linear regression models were used to compare groups, using the continuous variable as the dependent variable (age, height, weight, BMI), and the metabolic health as the independent variable (healthy, unhealthy). In these models, the beta-coefficient of metabolic health (independent variable) represents the difference between groups for the continuous (dependent) variable. A Student’s t test was then used to test whether the beta-coefficient differed from zero.

Data for categorical variables (lifestyle habits, education) were expressed as percentages [95% CI] (n applying sampling weights). Pearson Chi-square was used to test the unadjusted relationships between categorical variables and metabolic health.

Multivariate logistic regression models were used to compute odds ratio and 95% confidence intervals (OR [95% CI]) for the association between lifestyle habits and metabolic health (models 1 to 3). Analyses were conducted on the overall sample and also stratified by nutritional status (normal weight, overweight, obesity). Metabolic health was the outcome variable (healthy, unhealthy), considering the healthy phenotype as the reference. Lifestyle habits were the exposure variables, with their unhealthiest category considered as the reference–to highlight the effect of healthy habits. The lifestyle habits considered were smoking, alcohol intake, sedentary behavior, moderate-vigorous physical activity, fruits/vegetables consumption, and fish/seafood consumption. Age, sex, and BMI (in kg/m^2^) were considered as potential confounders, since they have been repeatedly reported to influence metabolic health [2]. Education–a surrogate of economic income– was also considered as a potential confounder, owing to the high economic inequality in Chile [18]. In all these regression models, continuous variables (e.g. age) were included as covariables, while categorical variables (e.g. sex) were included as factors.

As a sensitivity analysis, we repeated the multivariate logistic regression models using as outcome variable the presence–or absence–of metabolic syndrome. Therefore, subjects without metabolic syndrome included those with a healthy phenotype plus those with an intermediate phenotype; subjects with metabolic syndrome were the same as the metabolically unhealthy group. Also, we computed an additional multivariate logistic regression model adjusted for the confounding variables specific to the nutritional status (model 4).

The complex samples module of IBM® SPSS® Statistics v.25 was used. Specifically-calculated sampling weights were used, according to the survey’s complex sample [22]. *P* < 0.05 was considered statistically significant.

## Results

**Fig 1:**
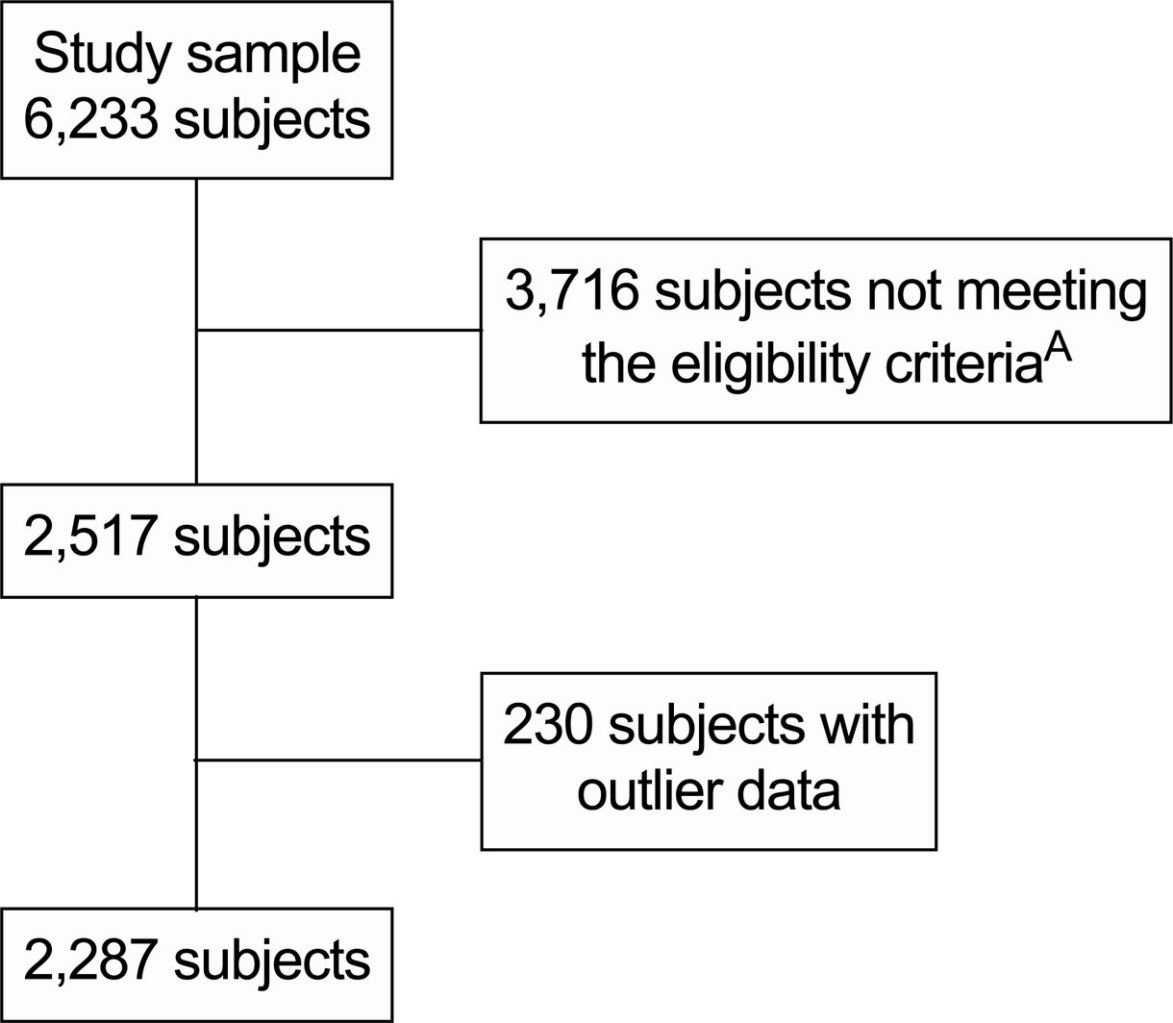
Flow diagram for selection of participants. ^A^18 to <65 years old, body mass index ≥18.5 kg/m^2^, along with complete data for blood pressure, circulating triglycerides, circulating glucose, circulating high-density lipoprotein cholesterol and waist circumference.

The survey included 6,233 participants. When considering our eligibility criteria and after discarding outlier data, 2,287 participants remained (848 healthy, 602 intermediate phenotype, 837 unhealthy; Fig 1). After applying the sampling weights, those 2,287 participants represented an estimated total [95% CI] of 10,137,832 [9,377,742–10,897,923] individuals in Chile.

Table 1 shows the general characteristics of the subjects included. Of note, there was about the same proportion of males and females, 78% had excess body weight, 36% had a metabolically unhealthy phenotype, and only 10% had less than 8 years of education. As for the lifestyle habits, 61% were non-smokers, 22% had a risky alcohol intake, 73% met the recommended levels of moderate-vigorous physical activity, and only 15% met the recommended fruits/vegetables consumption.

**Table 1:** General characteristics of the subjects.

	Mean or percentage	95% CI	n^A^
Age (years)	38.8	37.9–39.6	10,137,832
Females (%)	49.3	46.1–52.5	4,998,360
Height (m)	1.63	1.63–1.64	10,137,832
Weight (kg)	76.4	75.5–77.4	10,137,832
Nutritional status			
*Normal weight*, *18*.*5 to <25*.*0 kg/m*^*2*^ *(%)*	22.3	19.5 - 25.4	2,259,444
*Overweight*, *25*.*0 to <30*.*0 kg/m*^*2*^ *(%)*	44.5	40.6–48.4	4,506,673
*Obesity*, *>30*.*0 kg/m*^*2*^ *(%)*	33.3	30.0–36.7	3,371,714
Metabolic health^B^			
*Unhealhty*, *>2 risk factors (%)*	35.7	32.3–39.1	3,615,750
*Intermediate*, *2 risk factors (%)*	26.3	23.2–29.7	2,668,282
*Healhty*, *<2 risk factors*	38.0	34.6–41.5	3,853,798
Education^C^			
*<8 years (%)*	9.5	7.7–11.6	957,386
*8–12 years (%)*	58.8	54.5–63.0	5,937,583
*>12 years (%)*	31.7	27.8–35.8	3,197,615
Smoking			
*Current (%)*	39.3	36.0–42.7	3,984,050
*Former (%)*	22.3	19.7–25.1	2,259,013
*Never (%)*	38.4	35.2–41.8	3,894,768
Risky alcohol intake (%)^D^^,^^H^	22.3	19.3–25.6	2,262,623
Sedentary behavior (min/d)^E^	199	186–212	10,095,020
Moderate-vigorous physical activity ≥600 MET×min/wk (%)^F^	72.5	69.2–75.5	7,001,151
≥400 g/d of fruits/vegetables (%)^G^	14.8	12.1–17.9	1,482,939
Fish/seafood consumption			
*<1 time/month (%)*	32.4	29.0–35.9	3,280,369
*1 to <3 times/month (%)*	22.5	19.7–25.7	2,283,548
*4 times/month (%)*	35.2	31.5–39.0	3,565,746
*>4 times/month (%)*	9.9	8.2–12.1	1,008,167

^A^Applying sampling weights

^B^Considering the risk factors used to diagnose metabolic syndrome

^C^19

^D^1

^E^16

^F^95

^G^18 subjects excluded in the non-weighed sample

^H^Based on the AUDIT-C score: >3 points for women and >4 points for men. CI, confidence interval.

### Prevalence of metabolic phenotypes

**Fig 2:**
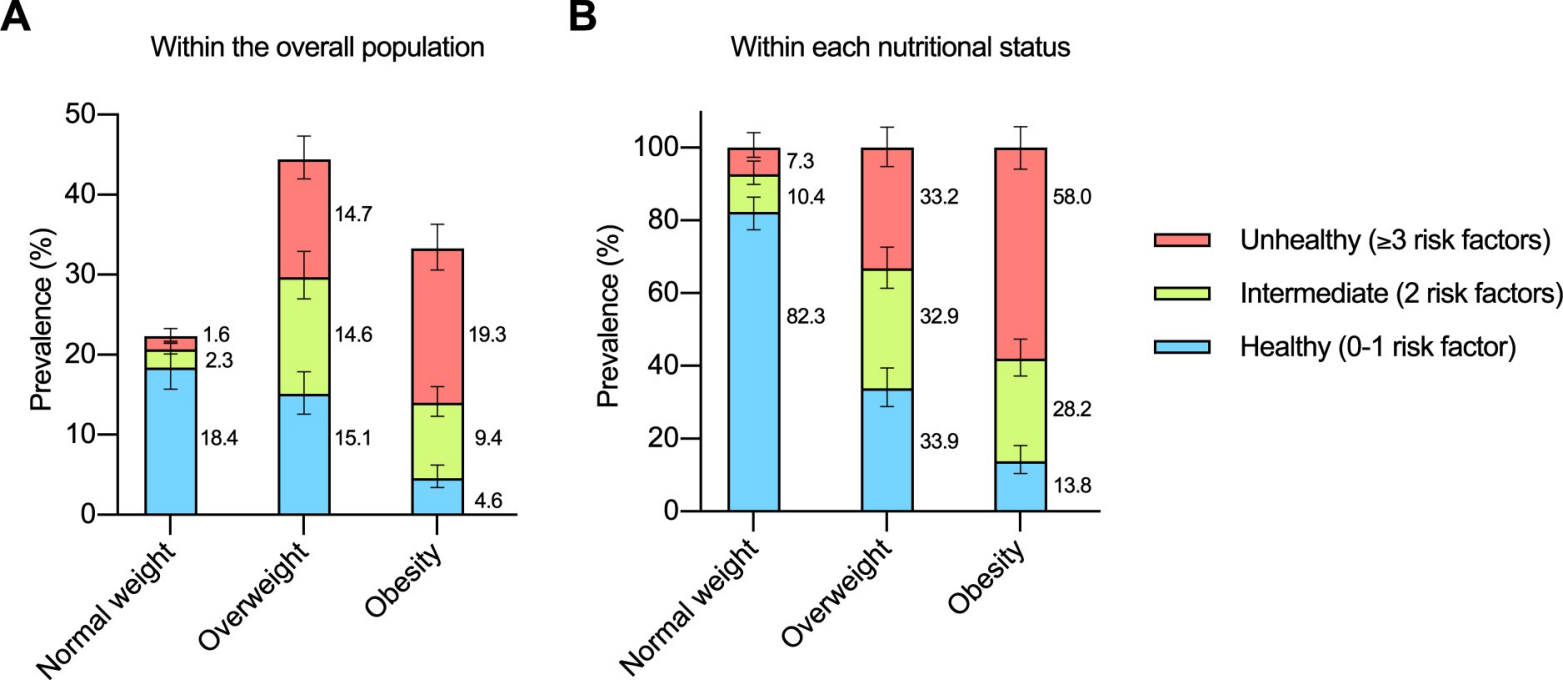
Prevalence of metabolic phenotypes in Chile. (A) Relative to the overall population (the overall population represents 100%). (B) According to the nutritional status (each nutritional status category represents 100%). Data are percentages with 95% confidence intervals.

Fig 2A shows the prevalence of metabolic phenotypes relative to the overall population. There were 1,860,637 [1,545,146–2,176,127] healthy subjects with normal weight, 234,051 [164,846–303,256] subjects with an intermediate phenotype and normal weight, 164,757 [88,442–241,071] unhealthy subjects with normal weight, 1,528,260 [1,235,281–1,821,239] healthy subjects with overweight, 1,483,785 [1,139,246–1,828,324] subjects with an intermediate phenotype and overweight, 1,494,628 [1,195,107–1,794,149] unhealthy subjects with overweight, 464,901 [321,990–607,813] healthy subjects with obesity, 950,447 [749,262–1,151,632] subjects with an intermediate phenotype and obesity, and 1,956,366 [1,665,454–2,247,278] unhealthy subjects with obesity.

Fig 2B shows the proportion of metabolic phenotypes within each nutritional status. The proportion of healthy individuals was progressively lower from normal weight to overweight to obesity, while unhealthy subjects had the opposite pattern. The metabolic phenotype (healthy, unhealthy) was related to nutritional status (normal weight, overweight, obesity). Thus, the higher the BMI, the higher the proportion of unhealthy subjects (*P* < 0.001 Pearson Chi-square).

### Comparisons between healthy and unhealthy subjects

Considering the overall sample (S1 Table), unhealthy subjects were older, heavier, and had higher BMI than their healthy counterparts. Also, the metabolic phenotype was related to sex, with a higher proportion of males among unhealthy subjects than among healthy subjects. The metabolic phenotype was related to education, highlighting a 2.1-fold higher proportion of healthy subjects with >12 years of education compared to unhealthy subjects (44.3% [38.2–50.6] vs. 20.9% [15.9–27.1], respectively).

Among subjects with normal weight, unhealthy subjects were older, heavier, and had higher BMI than their healthy counterparts. The metabolic phenotype was related to education, highlighting a 2.9-fold higher proportion of healthy subjects with >12 years of education compared to unhealthy subjects (47.0% [38.2–55.9] vs. 16.2% [4.4–44.9], respectively; Table 2).

**Table 2:** General characteristics and lifestyle habits according to nutritional status and metabolic health.

	Normal weight		Overweight		Obesity	
	Healthy	Unhealthy	Healthy	Unhealthy	Healthy	Unhealthy
Age (years)	31.6 [29.8–33.3] (1,860,636)	43.2 [37.7–48.7] (164,756)^C^	35.3 [33.4–37.1] (1,528,260)	44.9 [42.5–47.4] (1,494,628)^C^	35.9 [32.0–39.8] (464,901)	43.5 [41.8–45.1] (1,956,366)^B^
Sex						
*Female (%)*	47.9 [40.1–55.8] (890,895)	34.5 [19.0–54.1] (56,760)	54.1 [45.8–62.2] (826,246)	29.2 [22.9–36.5] (436,942)^C^	54.0 [38.2–69.0] (251,022)	52.1 [44.4–59.7] (1,019,726)
*Male (%)*	52.1 [44.2–59.9] (969,741)	65.5 [45.9–81.0] (107,996)	45.9 [37.8–54.2] (702,014)	70.8 [63.5–77.1] (1,057,685)^C^	46.0 [31.0–61.8] (213,878)	47.9 [40.3–55.6] (936,639)
Height (m)	1.64 [1.62–1.66] (1,860,636)	1.65 [1.62–1.69]	1.63 [1.61–1.64] (1,528,260)	1.66 [1.65–1.68] (1,494,628)^B^	1.63 [1.60–1.65] (464,901)	1.61 [1.60–1.63] (1,956,366)
Weight (kg)	60.6 [59.2–62.1] (1,860,636)	64.5 [61.5–67.4] (164,756)^A^	71.9 [70.4–73.4] (1,528,260)	77.4 [75.9–78.9] (1,494,628)^C^	87.4 [84.4–90.3] (464,901)	90.3 [88.4–92.3] (1,956,366)
Body mass index (kg/m^2^)	22.3 [22.0–22.6] (1,860,636)	23.3 [22.9–23.8] (164,756)^C^	26.9 [26.7–27.1] (1,528,260)	27.7 [27.4–28.0] (1,494,628)^C^	32.7 [32.0–33.5] (464,901)	34.5 [33.9–35.1] (1,956,366)^C^
Education						
*<8 years (%)*	6.9 [3.6–13.0] (128,453)	10.6 [4.0–25.1] (17,464)^A^	3.2 [1.7–5.9] (48,893)	12.6 [7.5–20.3] (186,956)^B^	6.2 [2.7–13.5] (28,734)	13.1 [9.0–18.6] (255,088)^C^
*8–12 years (%)*	46.1 [37.5–54.9] (854,998)	73.2 [49.0–88.6] (120,632)^A^	58.5 [49.1–67.3] (888,761)	66.8 [55.8–76.2] (993,219)^B^	40.6 [28.1–54.5] (188,693)	65.3 [57.7–72.2] (1,270,218)^C^
*>12 years (%)*	47.0 [38.2–55.9] (871,287)	16.2 [4.4–44.9] (26,659)^A^	38.3 [29.6–47.8] (582,134)	20.6 [12.5–32.1] (306,495)^B^	53.2 [39.2–66.7] (247,127)	21.6 [16.0–28.4] (419,384)^C^
Smoking						
*Current (%)*	36.8 [29.0–45.4] (685,160)	35.5 [17.6–58.7] (58,487)	37.9 [30.0–46.5] (579,265)	47.2 [37.3–57.3] (705,491)	35.6 [22.0–52.0] (165,480)	33.6 [27.1–40.9] (657,942)
*Former (%)*	16.4 [11.2–23.2] (304,582)	20.8 [7.7–45.2] (34,320)	23.9 [17.5–31.8] (365,604)	23.9 [17.1–32.5] (357,870)	36.5 [22.5–53.3] (169,733)	20.6 [15.7–26.5] (402,584)
*Never (%)*	46.8 [38.8–55.0] (870,893)	43.7 [23.2–66.6] (71,948)	38.2 [30.1–46.9] (583,390)	28.9 [21.6–37.4] (431,266)	27.9 [16.9–42.4] (129,686)	45.8 [38.5–53.3] (895,838)
Alcohol intake						
*AUDIT-C score >2 (%)*	43.2 [34.3–52.6] (804,537)	20.6 [9.3–39.5] (33,882)	31.6 [24.2–40.0] (482,509)	43.6 [35.0–52.6] (651,141)	36.1 [23.9–50.5] (166,224)	34.2 [27.1–42.0] (668,417)
*AUDIT-C score 2 (%)*	13.0 [8.8–18.7] (241,522)	17.5 [5.9–41.8] (28,815)	19.4 [11.7–30.5] (296,726)	16.3 [10.5–24.6] (244,030)	17.9 [8.4–34.1] (82,285)	14.5 [10.0–20.4] (282,972)
*AUDIT-C score 0 to 1 (%)*	43.8 [35.6–52.3] (814,577)	61.9 [39.7–80.1] (102,058)	49.0 [39.3–58.8] (749,024)	40.1 [32.1–48.7] (599,456)	46.0 [31.0–61.7] (211,727)	51.4 [43.9–58.8] (1,004,975)
Sedentary behavior						
*>300 min/d (%)*	24.0 [17.4–32.3] (445,451)	16.8 [4.7–45.3] (27,521)	15.2 [9.7–23.2] (232,346)	20.0 [12.2–31.0] (296,054)	22.5 [11.8–38.5] (104,432)	20.1 [14.7–26.9] (392,287)
*>150 to 300 min/d (%)*	32.6 [24.9–41.3] (603,923)	28.3 [12.2–52.9] (46,562)	39.2 [30.8–48.3] (597,404)	29.8 [20.6–40.9] (441,024)	18.3 [9.8–31.6] (85,007)	24.4 [18.4–31.6] (476,388)
*>60 to 150 min/d (%)*	17.5 [11.9–24.9] (324,199)	25.7 [11.6–47.6] (42,235)	17.1 [11.7–24.2] (259,879)	18.3 [13.0–25.1] (271,013)	34.3 [21.0–50.8] (159,551)	22.5 [16.8–29.3] (437,901)
*0 to 60 min/d (%)*	26.0 [19.3–33.9] (481,497)	29.2 [14.3–50.4] (47,977)	28.5 [21.9–36.1] (434,353)	31.9 [24.4–40.6] (472,989)	24.9 [15.2–38.1] (115,635)	33.0 [26.1–40.6] (642,573)
Moderate-vigorous physical activity						
*0 to 480 MET×min/wk (%)*	20.6 [15.2–27.3] (359,690)	19.5 [7.4–42.5] (31,241)	24.6 [17.1–34.4] (356,603)	23.6 [17.2–31.6] (335,932)	15.7 [8.0–28.6] (71,574)	31.8 [24.9–39.7] (591,773)
*>480 to 2*,*161 MET×min/wk (%)*	23.5 [17.1–31.3] (410,060)	23.4 [8.5–50.2] (37,547)	24.7 [17.4–33.8] (358,550)	23.7 [15.4–34.6] (336,681)	36.1 [21.4–54.1] (164,748)	22.7 [16.9–29.7] (422,190)
*>2*,*161 to 8*,*640 MET×min/wk (%)*	30.6 [22.7–39.7] (533,952)	20.5 [6.8–47.4] (32,791)	25.8 [17.7–36.1] (374,392)	24.4 [17.2–33.5] (347,040)	22.5 [12.6–37.0] (102,773)	27.2 [20.6–35.0] (505,551)
*>8*,*640 MET×min/wk (%)*	25.4 [18.7–33.5] (443,579)	36.6 [18.3–59.7] (58,589)	24.8 [17.9–33.3] (359,534)	28.3 [19.7–38.9] (402,751)	25.7 [14.8–40.8] (117,079)	18.3 [13.3–24.6] (339,878)
Fruits/vegetables consumption^D^						
*0 to 1*.*4 portions/d (%)*	28.6 [21.9–36.4] (524,840)	38.9 [18.9–63.5] (64,061)	29.1 [21.5–38.2] (431,131)	30.5 [22.6–39.9] (456,424)	23.3 [13.8–36.5] (106,779)	23.6 [18.3–29.8] (457,468)
*>1*.*4 to 2*.*1 portions/d (%)*	21.7 [15.5–29.4] (397,698)	19.2 [7.8–40.2] (31,630)	22.9 [15.6–32.5] (339,146)	21.9 [15.0–30.8] (326,988)	22.2 [11.8–37.8] (101,864)	30.0 [23.7–37.1] (581,646)
*>2*.*1 to 4*.*0 portions/d (%)*	28.9 [22.3–36.6] (530,869)	39.5 [20.4–62.4] (65,086)	29.1 [21.9–37.4] (430,021)	27.2 [19.8–36.1] (406,823)	34.2 [22.1–48.8] (157,038)	30.2 [23.5–37.9] (586,491)
*>4*.*0 portions/d (%)*	20.8 [14.3–29.3] (382,047)	2.4 [0.7–7.5] (3,977)	18.8 [12.9–26.7] (278,759)	20.4 [13.1–30.3] (304,391)	20.4 [9.4–38.6] (93,479)	16.3 [11.1–23.2] (315,458)
Fish/seafood consumption						
*<1 time/month (%)*	33.1 [25.6–41.7] (616,161)	45.2 [24.1–68.1] (74,395)	39.2 [29.9–49.2] (598,383)	29.3 [21.5–38.4] (437,248)	37.6 [23.8–53.9] (174,997)	25.6 [19.7–32.6] (500,671)
*1 to <3 times/month (%)*	19.2 [13.9–26.0] (357,049)	9.6 [3.8–22.4] (15,839)	18.2 [13.0–24.9] (278,214)	24.2 [16.4–34.2] (361,533)	29.3 [17.4–44.9] (136,059)	24.5 [18.4–31.9] (479,605)
*4 times/month (%)*	34.3 [26.2–43.5] (637,910)	35.5 [17.7–58.6] (58,506)	32.8 [24.7–42.1] (501,857)	38.1 [28.8–48.4] (569,525)	25.0 [13.2–42.1] (116,012)	38.9 [31.9–46.4] (760,905)
*>4 times/month (%)*	13.4 [8.5–20.6] (249,515)	9.7 [2.8–28.4] (16,015)	9.8 [5.8–16.2] (149,805)	8.5 [4.6–15.2] (126,321)	8.1 [3.2–19.1] (37,832)	11.0 [7.5–15.8] (215,184)

Data for continuous variables are mean [95% confidence interval] (n applying sampling weights), and for categorical variables are percentage [95% confidence interval] (n applying sampling weights).

^A^*P* < 0.05

^B^*P* < 0.01

^C^*P* < 0.001 vs. Healthy in the same nutritional status category.

^D^Portions of 80 g.

In the overweight category, unhealthy subjects were older, taller, heavier, and had higher BMI than their healthy counterparts. The metabolic phenotype was related to sex, with a higher proportion of males among the unhealthy subjects than among the healthy subjects. The metabolic phenotype was related to education, highlighting a 3.9-fold higher proportion of unhealthy subjects with <8 years of education compared to healthy subjects (12.6% [7.5–20.3] vs. 3.2% [1.7–5.9], respectively; Table 2).

Among the subjects with obesity, unhealthy subjects were older, and had higher BMI than their healthy counterparts. The metabolic phenotype was related to education, highlighting a 2.4-fold higher proportion of healthy subjects with>12 years of education compared to unhealthy subjects (53.2% [39.2–66.7] vs. 21.6% [16.0–28.4], respectively; Table 2).

Note that in all these unadjusted analyses, lifestyle habits were unrelated to the metabolic phenotype (*P* > 0.05, Pearson Chi-square test; Table 2).

### Association between lifestyle habits and metabolic phenotype

We computed the OR [95% CI] of having a metabolically unhealthy phenotype according to different categories of lifestyle habits. Note that the analyses were conducted considering the unhealthiest category of the lifestyle habit as the reference category. Logistic regression model 1 was unadjusted; model 2 was adjusted for age, sex, BMI (as a continuous variable, in kg/m^2^), and education; and model 3 was adjusted as model 2 plus all the remaining lifestyle habits. Since the nutritional status represents a range of BMI values, we adjusted models 2 and 3 for BMI in kg/m^2^ to remove the influence of BMI values within each range.

Considering the overall population, the highest quartile of moderate-vigorous physical activity was associated with reduced odds of having an unhealthy phenotype in model 2 (S2 Table). Also, former smoking was associated with reduced odds in models 2 and 3.

In subjects with normal weight (Table 3), the lowest tertile of alcohol intake was associated with increased odds of having an unhealthy phenotype in models 1 and 3. The highest quartile of fruits/vegetables consumption was associated with reduced odds of having an unhealthy phenotype in all models. Finally, fish/seafood consumption 1 to <3 times/month was associated with reduced odds of having an unhealthy phenotype in model 3.

**Table 3:** Odds ratio (OR) and 95% confidence intervals [95% CI] of having a metabolically unhealthy phenotype.

	Normal weight			Overweight			Obesity		
	Model 1	Model 2	Model 3	Model 1	Model 2	Model 3	Model 1	Model 2	Model 3
Smoking									
*Current*	1.00	1.00	1.00	1.00	1.00	1.00	1.00	1.00	1.00
*Former*	1.32 [0.35–4.90]	1.31 [0.34–5.04]	1.03 [0.19–5.40]	0.80 [0.40–1.57]	0.43 [0.19–0.98]	0.42 [0.17–1.00]	0.59 [0.24–1.43]	0.33 [0.14–0.80]	0.29 [0.10–0.80]
*Never*	0.96 [0.32–2.90]	1.24 [0.34–4.49]	0.75 [0.21–2.60]	0.60 [0.32–1.13]	0.48 [0.21–1.08]	0.49 [0.20–1.17]	1.73 [0.74–4.03]	1.31 [0.53–3.24]	1.40 [0.49–3.98]
Alcohol intake									
*AUDIT-C score >2*	1.00	1.00	1.00	1.00	1.00	1.00	1.00	1.00	1.00
*AUDIT-C score 2*	2.83 [0.65–12.18]	2.08 [0.38–11.35]	1.70 [0.29–9.71]	0.60 [0.26–1.39]	0.60 [0.24–1.48]	0.63 [0.27–1.50]	0.85 [0.31–2.31]	1.07 [0.45–2.57]	1.96 [0.69–5.54]
*AUDIT-C score 0 to 1*	2.97 [1.03–8.51]	3.16 [0.89–11.18]	3.48 [1.05–11.46]	0.59 [0.35–0.98]	0.91 [0.46–1.79]	0.84 [0.37–1.89]	1.18 [0.56–2.45]	1.14 [0.42–3.05]	1.57 [0.60–4.10]
Sedentary behavior									
*>300 min/d*	1.00	1.00	1.00	1.00	1.00	1.00	1.00	1.00	1.00
*>150 to 300 min/d*	1.24 [0.22–7.00]	0.63 [0.10–3.83]	0.64 [0.10–3.88]	0.57 [0.23–1.40]	0.43 [0.15–1.20]	0.50 [0.17–1.51]	1.49 [0.51–4.34]	1.36 [0.46–4.00]	0.95 [0.25–3.51]
*>60 to 150 min/d*	2.10 [0.40–11.08]	1.10 [0.23–5.13]	1.20 [0.24–6.02]	0.81 [0.34–1.95]	0.60 [0.22–1.64]	0.57 [0.19–1.64]	0.73 [0.26–2.04]	0.44 [0.16–1.22]	0.45 [0.14–1.49]
*0 to 60 min/d*	1.61 [0.33–7.72]	0.47 [0.09–2.27]	0.77 [0.15–3.93]	0.85 [0.37–1.93]	0.69 [0.26–1.79]	0.92 [0.34–2.49]	1.47 [0.58–3.76]	1.27 [0.47–3.43]	1.38 [0.49–3.91]
Moderate-vigorous physical activity									
*0 to 480 MET×min/wk*	1.00	1.00	1.00	1.00	1.00	1.00	1.00	1.00	1.00
*>480 to 2*,*161 MET×min/wk*	1.05 [0.22–4.84]	0.83 [0.11–5.96]	0.68 [0.11–4.22]	0.99 [0.44–2.21]	1.13 [0.44–2.85]	1.03 [0.41–2.59]	0.31 [0.10–0.88]	0.33 [0.10–1.05]	0.39 [0.13–1.10]
*>2*,*161 to 8*,*640 MET×min/wk*	0.70 [0.14–3.54]	0.72 [0.11–4.54]	1.46 [0.22–9.57]	0.98 [0.45–2.11]	0.73 [0.31–1.70]	0.65 [0.27–1.55]	0.59 [0.22–1.58]	0.47 [0.16–1.35]	0.80 [0.28–2.28]
*>8*,*640 MET×min/wk*	1.52 [0.41–5.61]	1.07 [0.24–4.79]	1.08 [0.21–5.55]	1.18 [0.58–2.42]	0.66 [0.29–1.48]	0.57 [0.23–1.41]	0.35 [0.12–0.97]	0.29 [0.09–0.91]	0.36 [0.11–1.19]
Fruits/vegetables consumption^A^									
*0 to 1*.*4 portions/d*	1.00	1.00	1.00	1.00	1.00	1.00	1.00	1.00	1.00
*>1*.*4 to 2*.*1 portions/d*	0.65 [0.17–2.44]	0.87 [0.19–4.02]	0.81 [0.21–3.03]	0.91 [0.42–1.93]	0.83 [0.35–1.94]	0.65 [0.27–1.53]	1.33 [0.55–3.22]	1.37 [0.53–3.54]	1.17 [0.42–3.24]
*>2*.*1 to 4*.*0 portions/d*	1.00 [0.30–3.30]	1.04 [0.23–4.63]	0.90 [0.21–3.83]	0.89 [0.45–1.74]	1.04 [0.43–2.49]	1.08 [0.47–2.49]	0.87 [0.39–1.93]	1.00 [0.41–2.42]	0.82 [0.28–2.38]
*>4*.*0 portions/d*	0.08 [0.01–0.37]	0.09 [0.01–0.48]	0.05 [0.01–0.40]	1.03 [0.47–2.25]	1.33 [0.51–3.47]	1.33 [0.48–3.69]	0.78 [0.26–2.37]	0.74 [0.25–2.19]	1.07 [0.35–3.28]
Fish/seafood consumption									
*<1 time/month*	1.00	1.00	1.00	1.00	1.00	1.00	1.00	1.00	1.00
*1 to <3 times/month*	0.36 [0.10–1.26]	0.29 [0.06–1.33]	0.16 [0.03–0.90]	1.77 [0.89–3.53]	1.71 [0.75–3.90]	1.24 [0.54–2.88]	1.23 [0.52–2.87]	1.19 [0.52–2.71]	1.02 [0.40–2.58]
*4 times/month*	0.76 [0.24–2.36]	0.80 [0.21–3.00]	0.69 [0.21–2.25]	1.55 [0.79–3.04]	1.46 [0.70–3.05]	1.18 [0.54–2.57]	2.29 [0.92–5.70]	2.20 [0.90–5.38]	2.14 [0.83–5.50]
*>4 times/month*	0.53 [0.11–2.50]	0.76 [0.12–4.64]	0.67 [0.06–7.11]	1.15 [0.45–2.89]	0.96 [0.30–3.09]	0.67 [0.14–3.11]	1.98 [0.66–5.92]	1.86 [0.53–6.48]	1.62 [0.44–5.92]

Model 1, not adjusted; Model 2, adjusted for age, sex, body mass index (as a continuous variable, in kg/m^2^), and education; Model 3, adjusted for age, sex, body mass index (as a continuous variable, in kg/m^2^), education, and all the remaining lifestyle habits shown in the table.

^A^Portions of 80 g.

In subjects with overweight (Table 3), former smoking was associated with reduced odds of having an unhealthy phenotype in model 2. Also, the lowest tertile of alcohol intake was associated with reduced odds of having an unhealthy phenotype in model 1.

In the obesity category (Table 3), the highest quartile of moderate-vigorous physical activity was associated with reduced odds of having an unhealthy phenotype in models 1 and 2, while the second quartile was associated with reduced odds in model 1. Finally, former smoking was associated with reduced odds of having an unhealthy phenotype in models 2 and 3.

### Sensitivity analyses

We determined the association between lifestyle habits and metabolic health, but considering as metabolically healthy those subjects with up to 2 risk factors (sensitivity analysis 1; S3 Table). Among subjects with normal weight, similar associations as those in the main analysis were observed, except for fish/seafood consumption. In the obesity category, the association between former smoking and metabolic health was also similar to the main analysis; a similar trend was observed for moderate-vigorous physical activity. Nevertheless, in subjects with overweight, smoking was not associated with metabolic health; moreover, the second quartile of sedentary behavior was associated with reduced odds of having a metabolically unhealthy phenotype in model 2. Among subjects with obesity, the second quartile of fruits/vegetables consumption was associated with elevated odds of having a metabolically unhealthy phenotype in models 1 and 2.

Finally, we determined the association between lifestyle habits and metabolic health adjusting for confounding variables specific to the nutritional status (sensitivity analysis 2). The results in Table 2 showed that, among subjects with normal weight or obesity, sex was not associated with metabolic health. Therefore, we computed the OR [95% CI] for having a metabolically unhealthy phenotype adjusted only for age, BMI (as a continuous variable, in kg/m^2^), and education (model 4; S4 Table). Again, the highest quartile of fruits/vegetables consumption was associated with reduced odds of having a metabolically unhealthy phenotype in subjects with normal weight. In subjects with obesity, former smoking was again associated with reduced odds of having a metabolically unhealthy phenotype.

## Discussion

Healthy lifestyle habits could prevent or reverse a metabolically unhealthy phenotype, thus reducing the risk of cardiovascular events and all-cause mortality. This is why the adoption of healthy habits represents a well-established public health recommendation [1]. Identification of the habits more strongly associated with metabolic health within groups of individuals would maximize the benefits of interventions. Herein, we found that the prevalence of a metabolically unhealthy phenotype was 7%, 33% and 58% among subjects with normal weight, overweight and obesity, respectively. Also, we observed associations between lifestyle habits and metabolic health that were specific to the nutritional status. In subjects with normal weight, consumption of fruits/vegetables was associated with reduced odds of having an unhealthy phenotype; and in subjects with obesity, moderate-vigorous physical activity was associated with reduced odds of having an unhealthy phenotype.

To characterize metabolic health, studies have considered diverse risk factors, including blood pressure, atherogenic dyslipidemia and insulin resistance, among others [2–6]. The number of risk factors required to classify subjects as metabolically healthy or unhealthy has also varied among studies [2,7,9,27]. These differences partly explain the variable prevalence of metabolically healthy and unhealthy subjects reported in various populations [2,3,28,29]. Herein we considered the risk factors that compose the metabolic syndrome, which are well-established risk factors for diabetes and cardiovascular disease [8,30,31]. We considered subjects having up to 1 of these risk factors as healthy, and those having ≥3 as unhealthy (people with metabolic syndrome). Using this classification, which excludes subjects with 2 risk factors, we intended to increase the chances to identify associations with lifestyle habits. This was supported by only one of our findings though. When subjects with up to 2 risk factors were considered as healthy (sensitivity analysis 1), the association between former smoking and metabolic health in subjects with overweight was not detected (S3 Table). Besides this difference, the results were essentially the same when considering as healthy those with up to 1 or those with up to 2 risk factors.

We found that the prevalence of a metabolically unhealthy phenotype in Chile was progressively higher going from subjects with normal weight (7%) to overweight (33%) to obesity (58%). This agrees with studies in other populations [7,9–12]. For instance, in the USA, the unhealthy phenotype was 9%, 34% and 61% in subjects with normal weight, overweight, and obesity, respectively [9]. Our findings thereby support the well-documented relationship between excess body weight and metabolic disturbances. Notably, we found that metabolically unhealthy subjects were older than their metabolically healthy counterparts. This can be explained because older subjects have had more time to accumulate risk factors. Indeed, evidence in subjects with obesity suggests that most metabolically healthy subjects become unhealthy in the long term [32]; additionally, older age has been shown to increase the risk of having a metabolically unhealthy phenotype [9]. Together, older subjects appear as a vulnerable group for a metabolically unhealthy phenotype within each nutritional status. We also observed that within a certain nutritional status (determined by a range of BMI values), the actual BMI value was higher in metabolically unhealthy subjects than in healthy subjects; nevertheless, this result was expected, as we considered elevated waist circumference to classify subjects as metabolically unhealthy, and waist circumference correlated directly with BMI in our data (Pearson r = 0.83, *P* < 0.001, n = 2,287).

In the overall population, higher moderate-vigorous physical activity and former smoking associated with reduced odds of having an unhealthy phenotype. This was expected based on previous evidence in other populations [12,33,34] and also in Chile [35]. Interestingly, some extra information arose when subjects were stratified according to the nutritional status. The association between former smoking and metabolic health appeared only in subjects with overweight or obesity. Similarly, the protective effect of moderate-vigorous physical activity was only evident in subjects with obesity. These observations support the idea that results obtained in the overall population (adjusted for BMI in kg/m^2^) reflect the most prevalent group (overweight/obese, 78% of our sample). But notably, in subjects with normal weight (22% of our sample), the highest quartile of fruits/vegetables consumption associated with reduced odds of having an unhealthy phenotype. The positive effect of former smoking (in obesity) and of fruits/vegetables consumption (in normal weight) remained significant in our sensitivity analyses, highlighting the strength of the associations. These analyses specific to the nutritional status highlighted how specific lifestyle habits associate with a metabolic phenotype in subjects with different nutritional statuses in Chile. These results support previous evidence that showed associations specific to the nutritional status in Korea [15], Spain [16], and the USA [17]. Focusing lifestyle interventions according to these results may enhance their effectiveness in Chile. Prospective studies should test such hypothesis.

Notably, the reduced odds of having an unhealthy phenotype in former–but not never–smokers in subjects with overweight or obesity, may result from a reverse causality association. Unhealthy individuals with excess body weight may have quitted smoking upon finding out some of their metabolic disturbances. Moreover, the observation that the lowest tertile of alcohol intake was associated with elevated odds of having a metabolically unhealthy phenotype in subjects with normal weight was unexpected. Note, however, that this association appeared in models 1 and 3, and may result from lack of adjustment (model 1) and over-adjustment (model 3); in contrast, alcohol intake did not associate with metabolic health in model 2 or in the model with adjustments specific to the nutritional status (sensitivity analysis 2).

The main limitation of our study is that questionnaires were used to estimate lifestyle habits. The main issue with diet questionnaires is the under-report, whereas for physical-activity questionnaires it is the over-report [36]. We tried to minimize this bias by categorizing the variables into quartiles or tertiles, to compare the extremes of each lifestyle habit (e.g. quartile 1 vs. quartile 4). And although some inaccuracy remains, questionnaires are currently the most used tool for population-based studies. It is also worth noting that the inaccuracy may indeed weaken–not strengthen–the associations between lifestyle habits and metabolic health, as shown for the association between physical activity and adiposity [37].

## Conclusions

We have shown that about one third of the Chilean population manifests a metabolically unhealthy phenotype, but this prevalence varies across nutritional statuses. Regarding lifestyle habits, our findings are consistent with the well-known benefits that adopting healthy lifestyles habits has on chronic diseases [38–40]. Of note, we have shown that specific lifestyle habits associate with metabolic health across nutritional statuses. Fruits/vegetables consumption–in subjects with normal weight–, and high levels of physical activity–in subjects with obesity–showed associations with reduced risk of having a metabolically unhealthy phenotype. This information may serve to complement public health interventions with recommendations specific to the nutritional status. For instance, by emphasizing the consumption of fruits and vegetables in subjects with normal weight, and physical activity in subjects with obesity.

## Supporting information

S1 File STROBE checklist for cross-sectional studies. (DOCX)

S1 Table General characteristics and lifestyle habits in the overall sample. (DOCX)

S2 Table Odds ratio (OR) and 95% confidence intervals [95% CI] of having a metabolically unhealthy phenotype. (DOCX)

S3 Table Odds ratio (OR) and 95% confidence intervals [95% CI] of having a metabolically unhealthy phenotype (sensitivity analysis 1). (DOCX)

S4 Table Odds ratio (OR) and 95% confidence intervals [95% CI] of having a metabolically unhealthy phenotype (sensitivity analysis 2). (DOCX)
