# Supplementary material for: Metabolic health and its association with lifestyle habits according to nutritional status in Chile: A cross-sectional study from the National Health Survey 2016-2017
Source: PLoS One. 2020 Jul 22;15(7):e0236451. doi: 10.1371/journal.pone.0236451 (PMC7375524; doi:10.1371/journal.pone.0236451)
Supplement: S1 Table — (DOCX) [file pone.0236451.s002.docx]

| **S1 Table. General characteristics and lifestyle habits in the overall sample.** | | | |
| --- | --- | --- | --- |
|  | **All subjects** | | |
|  | **Healthy** | **Unhealthy** | |
| Age (years) | 33.6 [32.3 - 34.8] (3,853,798) | | 44.1 [42.7 - 45.4] (3,615,750)^C^ |
| Sex |  | |  |
| *Female (%)* | 51.1 [45.8 - 56.3] (1,968,164) | | 41.9 [36.5 - 47.4] (1,513,428)^A^ |
| *Male (%)* | 48.9 [43.7 - 54.2] (1,885,634) | | 58.1 [52.6 - 63.5] (2,102,321)^A^ |
| Height (m) | 1.63 [1.62 - 1.64] (3,853,798) | | 1.64 [1.62 - 1.65] (3,615,750) |
| Weight (kg) | 68.3 [66.9 - 69.8] (3,853,798) | | 83.8 [82.4 - 85.2] (3,615,750)^C^ |
| Body mass index (kg/m^2^) | 25.4 [25.0 - 25.9] (3,853,798) | | 31.2 [30.6 - 31.7] (3,615,750)^C^ |
| Education |  | |  |
| *<8 years (%)* | 5.4 [3.4 - 8.3] (206,081) | | 12.8 [9.5 - 17.0] (459,508)^C^ |
| *8-12 years (%)* | 50.3 [44.1 - 56.6] (1,932,454) | | 66.3 [60.0 - 72.1] (2,384,070)^C^ |
| *>12 years (%)* | 44.3 [38.2 - 50.6] (1,700,549) | | 20.9 [15.9 - 27.1] (752,539)^C^ |
| Smoking |  | |  |
| *Current (%)* | 37.1 [31.8 - 42.8] (1,429,907) | | 39.3 [33.7 - 45.2] (1,421,922) |
| *Former (%)* | 21.8 [17.5 - 26.9] (839,920) | | 22.0 [18.0 - 26.6] (794,775) |
| *Never (%)* | 41.1 [35.7 - 46.7] (1,583,971) | | 38.7 [33.1 - 44.6] (1,399,052) |
| Alcohol intake |  | |  |
| *AUDIT-C score >2 (%)* | 37.8 [32.0 - 43.9] (1,453,271) | | 37.4 [32.1 - 43.1] (1,353,441) |
| *AUDIT-C score 2 (%)* | 16.1 [12.0 - 21.2] (620,534) | | 15.4 [11.8 - 19.8] (555,817) |
| *AUDIT-C score 0 to 1 (%)* | 46.1 [40.4 - 52.0] (1,775,329) | | 47.2 [41.7 - 52.7] (1,706,491) |
| Sedentary behavior |  | |  |
| *>300 min/d (%)* | 20.4 [15.8 - 25.9] (782,230) | | 19.9 [15.2 - 25.7] (715,863) |
| *>150 to 300 min/d (%)* | 33.5 [28.2 - 39.2] (1,286,335) | | 26.8 [21.6 - 32.8] (963,976) |
| *>60 to 150 min/d (%)* | 19.3 [15.3 - 24.2] (743,630) | | 20.9 [16.9 - 25.6] (751,150) |
| *0 to 60 min/d (%)* | 26.8 [22.2 - 32.0] (1,031,486) | | 32.4 [27.3 - 37.8] (1,163,540) |
| Moderate-vigorous physical activity |  | |  |
| *0 to 480 MET×min/wk (%)* | 21.6 [17.3 - 26.5] (787,867) | | 27.9 [23.0 - 33.3] (958,946) |
| *>480 to 2,161 MET×min/wk (%)* | 25.6 [20.4 - 31.5] (933,359) | | 23.1 [18.0 - 29.2] (796,419) |
| *>2,161 to 8,640 MET×min/wk (%)* | 27.7 [22.3 - 33.8] (1,011,117) | | 25.7 [20.5 - 31.8] (885,383) |
| *>8,640 MET×min/wk (%)* | 25.2 [20.6 - 30.4] (920,193) | | 23.3 [18.4 - 20.0] (801,219) |
| Fruits/vegetables consumption^D^ |  | |  |
| *0 to 1.4 portions/d (%)* | 28.2 [23.3 - 33.6] (1,062,750) | | 27.2 [22.3 - 32.6] (977,953) |
| *>1.4 to 2.1 portions/d (%)* | 22.2 [17.7 - 27.5] (838,709) | | 26.1 [21.4 - 31.5] (940,266) |
| *>2.1 to 4.0 portions/d (%)* | 29.6 [24.9 - 34.8] (1,117,930) | | 29.4 [24.5 - 34.9] (1,058,401) |
| *>4.0 portions/d (%)* | 20.0 [15.4 - 25.6] (754,286) | | 17.3 [12.8 - 23.0] (623,827) |
| Fish/seafood consumption |  | |  |
| *<1 time/month (%)* | 36.1 [30.7 - 41.8] (1,389,541) | | 28.0 [23.2 - 33.4] (1,012,315) |
| *1 to <3 times/month (%)* | 20.0 [16.2 - 24.4] (771,323) | | 23.7 [18.9 - 29.3] (856,977) |
| *4 times/month (%)* | 32.6 [27.0 - 38.8] (1,255,780) | | 38.4 [33.0 - 44.2] (1,388,937) |
| *>4 times/month (%)* | 11.3 [8.2 - 15.5] (437,153) | | 9.9 [7.2 - 13.4] (357,520) |
| Data for continuous variables are mean [95% confidence interval] (n applying sampling weights), and for categorical variables are percentage [95% confidence interval] (n applying sampling weights). ^A^*P* < 0.05, ^B^*P* < 0.01, ^C^*P* < 0.001 vs. Healthy. ^D^Portions of 80 g. | | | |
