# Supplementary material for: Metabolic health and its association with lifestyle habits according to nutritional status in Chile: A cross-sectional study from the National Health Survey 2016-2017
Source: PLoS One. 2020 Jul 22;15(7):e0236451. doi: 10.1371/journal.pone.0236451 (PMC7375524; doi:10.1371/journal.pone.0236451)
Supplement: S2 Table — (DOCX) [file pone.0236451.s003.docx]

| **S2 Table. Odds ratio (OR) and 95% confidence intervals [95% CI] of having a metabolically unhealthy phenotype.** | | | |
| --- | --- | --- | --- |
|  | **All subjects** | | |
|  | **Model 1** | **Model 2** | **Model 3** |
| Smoking |  |  |  |
| *Current* | 1.00 | 1.00 | 1.00 |
| *Former* | 0.95 [0.62 - 1.45] | 0.43 [0.23 - 0.80] | 0.42 [0.21 - 0.81] |
| *Never* | 0.88 [0.60 - 1.30] | 0.75 [0.43 - 1.30] | 0.75 [0.41 - 1.35] |
| Alcohol intake |  |  |  |
| *AUDIT-C score >2* | 1.00 | 1.00 | 1.00 |
| *AUDIT-C score 2* | 0.96 [0.58 - 1.59] | 0.88 [0.48 - 1.62] | 1.06 [0.57 - 1.96] |
| *AUDIT-C score 0 to 1* | 1.03 [0.73 - 1.45] | 1.03 [0.64 - 1.66] | 1.05 [0.61 - 1.81] |
| Sedentary behavior |  |  |  |
| *>300 min/d* | 1.00 | 1.00 | 1.00 |
| *>150 to 300 min/d* | 0.81 [0.48 - 1.39] | 0.66 [0.31 - 1.38] | 0.74 [0.31 - 1.74] |
| *>60 to 150 min/d* | 1.10 [0.64 - 1.89] | 0.52 [0.23 - 1.13] | 0.59 [0.25 - 1.36] |
| *0 to 60 min/d* | 1.23 [0.75 - 2.01] | 0.77 [0.38 - 1.54] | 0.99 [0.47 - 2.08] |
| Moderate-vigorous physical activity |  |  |  |
| *0 to 480 MET×min/wk* | 1.00 | 1.00 | 1.00 |
| *>480 to 2,161 MET×min/wk* | 0.70 [0.43 - 1.13] | 0.72 [0.37 - 1.42] | 0.67 [0.34 - 1.31] |
| *>2,161 to 8,640 MET×min/wk* | 0.71 [0.45 - 1.13] | 0.64 [0.35 - 1.15] | 0.62 [0.34 - 1.14] |
| *>8,640 MET×min/wk* | 0.71 [0.45 - 1.12] | 0.55 [0.31 - 0.97] | 0.56 [0.31 - 1.03] |
| Fruits/vegetables consumption^A^ |  |  |  |
| *0 to 1.4 portions/d* | 1.00 | 1.00 | 1.00 |
| *>1.4 to 2.1 portions/d* | 1.21 [0.79 - 1.87] | 0.95 [0.53 - 1.70] | 0.74 [0.40 - 1.38] |
| *>2.1 to 4.0 portions/d* | 1.02 [0.68 - 1.55] | 1.01 [0.55 - 1.84] | 0.96 [0.51 - 1.84] |
| *>4.0 portions/d* | 0.89 [0.54 - 1.48] | 0.97 [0.48 - 1.95] | 0.90 [0.43 - 1.88] |
| Fish/seafood consumption |  |  |  |
| *<1 time/month* | 1.00 | 1.00 | 1.00 |
| *1 to <3 times/month* | 1.52 [1.00 - 2.31] | 1.22 [0.70 - 2.11] | 1.05 [0.61 - 1.79] |
| *4 times/month* | 1.51 [1.00 - 2.28] | 1.52 [0.91 - 2.55] | 1.46 [0.85 - 2.52] |
| *>4 times/month* | 1.12 [0.65 - 1.92] | 1.19 [0.52 - 2.70] | 1.04 [0.37 - 2.88 |
| Model 1, not adjusted; Model 2, adjusted for age, sex, body mass index (as a continuous variable, in kg/m^2^), and education; Model 3, adjusted for age, sex, body mass index (as a continuous variable, in kg/m^2^), education, and all the remaining lifestyle habits shown in the table. ^A^Portions of 80 g. | | | |
