# Supplementary material for: Metabolic health and its association with lifestyle habits according to nutritional status in Chile: A cross-sectional study from the National Health Survey 2016-2017
Source: PLoS One. 2020 Jul 22;15(7):e0236451. doi: 10.1371/journal.pone.0236451 (PMC7375524; doi:10.1371/journal.pone.0236451)
Supplement: S3 Table — (DOCX) [file pone.0236451.s004.docx]

| **S3 Table. Odds ratio (OR) and 95% confidence intervals [95% CI] of having a metabolically unhealthy phenotype (sensitivity analysis 1).** | | | | | | | | | |
| --- | --- | --- | --- | --- | --- | --- | --- | --- | --- |
|  | **Normal weight** | | | **Overweight** | | | **Obesity** | | |
|  | **Model 1** | **Model 2** | **Model 3** | **Model 1** | **Model 2** | **Model 3** | **Model 1** | **Model 2** | **Model 3** |
| Smoking |  |  |  |  |  |  |  |  |  |
| *Current* | 1.00 | 1.00 | 1.00 | 1.00 | 1.00 | 1.00 | 1.00 | 1.00 | 1.00 |
| *Former* | 1.45 [0.39 - 5.34] | 1.57 [0.43 - 5.69] | 1.10 [0.25 - 4.84] | 0.84 [0.46 - 1.53] | 0.56 [0.27 - 1.17] | 0.55 [0.26 - 1.16] | 0.80 [0.46 - 1.41] | 0.52 [0.29 - 0.95] | 0.47 [0.25 - 0.86] |
| *Never* | 1.05 [0.35 - 3.11] | 1.25 [0.38 - 4.13] | 0.71 [0.22 - 2.20] | 0.68 [0.39 - 1.19] | 0.73 [0.39 - 1.35] | 0.66 [0.33 - 1.29] | 1.58 [0.93 - 2.68] | 1.32 [0.75 - 2.33] | 1.23 [0.69 - 2.19] |
| Alcohol intake |  |  |  |  |  |  |  |  |  |
| *AUDIT-C score >2* | 1.00 | 1.00 | 1.00 | 1.00 | 1.00 | 1.00 | 1.00 | 1.00 | 1.00 |
| *AUDIT-C score 2* | 2.82 [0.66 - 11.92] | 2.68 [0.56 - 12.74] | 2.38 [0.49 - 11.58] | 0.88 [0.43 - 1.79] | 0.97 [0.43 - 2.17] | 1.03 [0.48 - 2.22] | 0.84 [0.42 - 1.67] | 0.84 [0.41 - 1.71] | 0.86 [0.41 - 1.82] |
| *AUDIT-C score 0 to 1* | 2.86 [1.01 - 8.09] | 3.25 [0.97 - 10.84] | 3.64 [1.08 - 12.27] | 0.67 [0.42 - 1.06] | 1.04 [0.60 - 1.80] | 1.08 [0.59 - 1.98] | 0.90 [0.55 - 1.47] | 0.82 [0.43 - 1.55] | 0.83 [0.46 - 1.50] |
| Sedentary behavior |  |  |  |  |  |  |  |  |  |
| *>300 min/d* | 1.00 | 1.00 | 1.00 | 1.00 | 1.00 | 1.00 | 1.00 | 1.00 | 1.00 |
| *>150 to 300 min/d* | 1.32 [0.23 - 7.32] | 0.70 [0.13 - 3.73] | 0.68 [0.13 - 3.41] | 0.69 [0.31 - 1.53] | 0.53 [0.23 - 1.21] | 0.55 [0.23 - 1.30] | 1.00 [0.49 - 2.04] | 1.12 [0.56 - 2.23] | 1.10 [0.53 - 2.27] |
| *>60 to 150 min/d* | 2.06 [0.40 - 10.65] | 1.01 [0.23 - 4.39] | 1.12 [0.29 - 4.39] | 0.69 [0.31 -1.49] | 0.38 [0.16 - 0.89] | 0.37 [0.14 - 0.92] | 0.85 [0.43 - 1.71] | 0.75 [0.38 - 1.47] | 0.80 [0.40 - 1.61] |
| *0 to 60 min/d* | 1.56 [0.33 - 7.39] | 0.49 [0.10 - 2.26] | 0.76 [0.17 - 3.26] | 0.85 [0.41 - 1.76] | 0.52 [0.23 - 1.19] | 0.57 [0.23 - 1.41] | 1.36 [0.72 - 2.56] | 1.59 [0.82 - 3.08] | 1.93 [0.95 - 3.90] |
| Moderate-vigorous physical activity |  |  |  |  |  |  |  |  |  |
| *0 to 480 MET×min/wk* | 1.00 | 1.00 | 1.00 | 1.00 | 1.00 | 1.00 | 1.00 | 1.00 | 1.00 |
| *>480 to 2,161 MET×min/wk* | 1.10 [0.24 - 4.99] | 0.91 [0.15 - 5.43] | 0.79 [0.14 - 4.50] | 1.01 [0.49 - 2.06] | 1.08 [0.47 - 2.45] | 1.16 [0.54 - 2.49] | 0.64 [0.34 - 1.19] | 0.74 [0.37 - 1.45] | 0.74 [0.39 - 1.41] |
| *>2,161 to 8,640 MET×min/wk* | 0.77 [0.15 - 3.80] | 0.82 [0.14 - 4.86] | 1.73 [0.27 - 11.08] | 1.15 [0.63 - 2.12] | 1.04 [0.51 - 2.10] | 1.05 [0.54 - 2.02] | 0.99 [0.55 - 1.75] | 0.94 [0.50 - 1.78] | 1.19 [0.63 - 2.23] |
| *>8,640 MET×min/wk* | 1.74 [0.48 - 6.34] | 1.42 [0.34 - 5.97] | 1.63 [0.33 - 8.05] | 1.27 [0.65 - 2.47] | 0.98 [0.48 - 2.00] | 1.15 [0.56 - 2.33] | 0.51 [0.26 - 1.00] | 0.49 [0.24 - 1.00] | 0.49 [0.24 - 1.00] |
| Fruits/vegetables consumption^A^ |  |  |  |  |  |  |  |  |  |
| *0 to 1.4 portions/d* | 1.00 | 1.00 | 1.00 | 1.00 | 1.00 | 1.00 | 1.00 | 1.00 | 1.00 |
| *>1.4 to 2.1 portions/d* | 0.63 [0.17 - 2.32] | 0.74 [0.17 - 3.21] | 0.76 [0.21 - 2.78] | 1.17 [0.61 - 2.24] | 1.33 [0.66 - 2.67] | 1.02 [0.49 - 2.12] | 1.99 [1.15 - 3.44] | 1.90 [1.07 - 3.36] | 1.77 [0.99 - 3.17] |
| *>2.1 to 4.0 portions/d* | 0.93 [0.28 - 3.05] | 0.96 [0.23 - 3.96] | 0.89 [0.19 - 4.02] | 1.02 [0.56 - 1.86] | 1.24 [0.62 - 2.49] | 1.26 [0.63 - 2.53] | 1.31 [0.74 - 2.33] | 1.27 [0.68 - 2.35] | 1.18 [0.62 - 2.22] |
| *>4.0 portions/d* | 0.08 [0.01 - 0.36] | 0.09 [0.02 - 0.47] | 0.07 [0.01 - 0.45] | 1.16 [0.55 - 2.44] | 1.82 [0.79 - 4.18] | 1.78 [0.71 - 4.47] | 1.09 [0.52 - 2.26] | 1.06 [0.50 - 2.24] | 1.23 [0.59 - 2.57] |
| Fish/seafood consumption |  |  |  |  |  |  |  |  |  |
| *<1 time/month* | 1.00 | 1.00 | 1.00 | 1.00 | 1.00 | 1.00 | 1.00 | 1.00 | 1.00 |
| *1 to <3 times/month* | 0.36 [0.10 - 1.23] | 0.36 [0.09 - 1.45] | 0.19 [0.03 - 1.01] | 1.29 [0.67 - 2.46] | 0.97 [0.44 - 2.14] | 0.83 [0.37 - 1.83] | 1.43 [0.79 - 2.61] | 1.29 [0.70 - 2.38] | 1.08 [0.57 - 2.02] |
| *4 times/month* | 0.80 [0.26 - 2.46] | 0.89 [0.25 - 3.13] | 0.63 [0.20 - 1.99] | 1.30 [0.71 - 2.37] | 1.12 [0.58 - 2.18] | 1.02 [0.52 - 1.98] | 1.78 [1.01 - 3.11] | 1.55 [0.87 - 2.75] | 1.33 [0.73 - 2.41] |
| *>4 times/month* | 0.53 [0.11 - 2.44] | 0.66 [0.12 - 3.64] | 0.59 [0.07 - 4.93] | 1.35 [0.59 - 3.04] | 1.09 [0.42 - 2.77] | 0.97 [0.32 - 2.97] | 1.57 [0.77 - 3.22] | 1.27 [0.59 - 2.76] | 1.03 [0.48 - 2.21] |
| Model 1, not adjusted; Model 2, adjusted for age, sex, body mass index (as a continuous variable, in kg/m^2^), and education; Model 3, adjusted for age, sex, body mass index (as a continuous variable, in kg/m^2^), education, and all the remaining lifestyle habits shown in the table. ^A^Portions of 80 g. | | | | | | | | | |
