# Supplementary material for: Metabolic health and its association with lifestyle habits according to nutritional status in Chile: A cross-sectional study from the National Health Survey 2016-2017
Source: PLoS One. 2020 Jul 22;15(7):e0236451. doi: 10.1371/journal.pone.0236451 (PMC7375524; doi:10.1371/journal.pone.0236451)
Supplement: S4 Table — (DOCX) [file pone.0236451.s005.docx]

| **S4 Table. Odds ratio (OR) and 95% confidence intervals [95% CI] of having a metabolically unhealthy phenotype (sensitivity analysis 2).** | | |
| --- | --- | --- |
|  | **Normal weight** | **Obesity** |
|  | **Model 4** | **Model 4** |
| Smoking |  |  |
| *Current* | 1.00 | 1.00 |
| *Former* | 1.30 [0.33 - 5.07] | 0.34 [0.14 - 0.86] |
| *Never* | 1.01 [0.28 - 3.61] | 1.26 [0.52 - 3.05] |
| Alcohol intake |  |  |
| *AUDIT-C score >2* | 1.00 | 1.00 |
| *AUDIT-C score 2* | 1.96 [0.44 - 8.75] | 1.01 [0.41 - 2.47] |
| *AUDIT-C score 0 to 1* | 2.34 [0.76 - 7.18] | 1.00 [0.44 - 2.23] |
| Sedentary behavior |  |  |
| *>300 min/d* | 1.00 | 1.00 |
| *>150 to 300 min/d* | 0.74 [0.13 - 4.16] | 1.34 [0.45 - 3.96] |
| *>60 to 150 min/d* | 1.14 [0.25 - 5.24] | 0.43 [0.16 - 1.12] |
| *0 to 60 min/d* | 0.49 [0.10 - 2.31] | 1.22 [0.46 - 3.23] |
| Moderate-vigorous physical activity |  |  |
| *0 to 480 MET×min/wk* | 1.00 | 1.00 |
| *>480 to 2,161 MET×min/wk* | 1.06 [0.16 - 6.86] | 0.38 [0.12 - 1.19] |
| *>2,161 to 8,640 MET×min/wk* | 0.77 [0.11 - 5.07] | 0.51 [0.17 - 1.49] |
| *>8,640 MET×min/wk* | 1.36 [0.33 - 5.50] | 0.37 [0.12 - 1.17] |
| Fruits/vegetables consumption^A^ |  |  |
| *0 to 1.4 portions/d* | 1.00 | 1.00 |
| *>1.4 to 2.1 portions/d* | 0.84 [0.18 - 3.92] | 1.38 [0.53 - 3.59] |
| *>2.1 to 4.0 portions/d* | 0.99 [0.21 - 4.52] | 0.97 [0.40 - 2.33] |
| *>4.0 portions/d* | 0.07 [0.01 - 0.41] | 0.73 [0.25 - 2.12] |
| Fish/seafood consumption |  |  |
| *<1 time/month* | 1.00 | 1.00 |
| *1 to <3 times/month* | 0.31 [0.07 - 1.29] | 1.18 [0.51 - 2.72] |
| *4 times/month* | 0.77 [0.20 - 2.95] | 2.25 [0.91 - 5.54] |
| *>4 times/month* | 0.76 [0.14 - 4.18] | 1.83 [0.53 - 6.32] |
| Model 4, adjusted for age, body mass index (as a continuous variable, in kg/m^2^), and education. ^A^Portions of 80 g. | | |
